# Effect of anti‐CGRP‐targeted therapy on migraine aura: Results of an observational case series study

**DOI:** 10.1111/cns.14595

**Published:** 2024-02-08

**Authors:** Elena Cresta, Alessia Bellotti, Giovanni Rinaldi, Ilenia Corbelli, Paola Sarchielli

**Affiliations:** ^1^ Neurologic Clinic University of Perugia Perugia Italy

**Keywords:** anti‐CGRP monoclonal antibodies, calcitonin gene‐related peptide (CGRP), cortical spreading depression, migraine aura

## Abstract

**Introduction:**

Limited clinical evidence is available regarding the potential effectiveness of anti‐CGRP monoclonal antibodies for the preventive treatment of migraine with aura.

**Aim of the Study:**

This observational study involved a series of migraine patients affected by either migraine with or without aura, who were investigated for any changes in their frequencies and their migraine aura attack characteristics observed during treatment with anti‐CGRP Mabs over a 1‐year period.

**Patients and Methods:**

Twelve migraine patients were included, seven of whom were treated with erenumab, 2 with fremanezumab, and 3 with galcanezumab. Clinical data were collected at baseline, which were defined as 3 months prior to the initiation of treatment, and thereafter at each trimester, over the 1‐year treatment period. The parameters included the number of headache and migraine days/month, the frequency of aura episodes, the number of days with acute drug intakes/month, and the scores from the migraine disability status scale (MIDAS), and the Headache Impact Test 6 (HIT‐6).

**Results:**

Anti‐CGRP Mbs antibodies induced significant decreases in mean headache and migraine without aura days per month, the number of days with medication intake, as well as MIDAS and HIT‐6 scores (*p* < 0.0001). In contrast, the anti‐CGRP Mab treatment did not appear to impact the frequency of migraine with aura attacks but seemed to reduce both the intensity and the duration of headache phases of migraine aura. Furthermore, some migraine patients referred to having aura attacks without headache over the course of the treatment period.

**Conclusions:**

Based on the above findings, we hypothesize that anti‐CGRP Mabs did not influence neuronal and vascular events related to cortical spreading depression (CSD) which is considered the pathophysiological substrate of aura. Conversely, these antibodies are able to counteract, via their peripheral mechanisms of action, the sensitization of the trigemino‐vascular pathway which is triggered by CSD. This aforementioned might explain why in our patients, migraine aura attacks remained unchanged in their frequencies, but the headache phases were either reduced or absent.

## Graphical abstract

This observational study involving a series of migraine patients affected by both migraine with and without aura reveals that anti‐CGRP antibodies induced a significant decrease in headache frequency of migraine without aura but not of migraine with aura attacks. Conversely, a reduction of the frequency and intensity of the headache phase of migraine aura and sometimes aura episodes without headache were recorded.

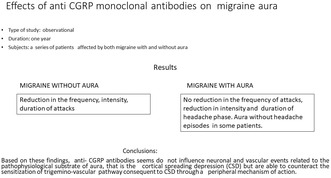

## 1 INTRODUCTION

Migraine aura refers to focal and reversible neurological symptoms occurring, usually before but sometimes during the headache phase. In fact, in about 90% of patients fully reversible symptoms are visual, followed by sensory disturbances and speech /language disturbances. Aura symptoms rarely suggest a brainstem or retinal origin. Motor symptoms are specific to sporadic or familial forms of hemiplegic migraine which is classified as a separate clinical entity due to its genetic basis and pathophysiological peculiarities.^1^

Typically, at least one aura symptom spreads gradually over ≥5 min, lasts from 5 to 60 min, and is unilateral and positive, whereas in the case of two or more symptoms, these will occur in succession. Headache following or accompanying migraine aura sometimes does not satisfy migraine without aura criteria and is therefore referred to the patient as being dull, having slight intensity, and without any associated symptoms. In other cases, aura is not followed by headache especially in patients over 50 years of age who refer aura in the absence of headache in 37% of cases, compared with patients under 50 years who refer aura in only 4% of cases.^2^ From 25% to 30% of migraine patients report aura symptoms preceding or accompanying their headaches.^3^ These patients are classified as being affected by both migraine with and without aura.

Cortical spreading depression (CSD) is considered the pathophysiological substrate of migraine aura.^4^, ^5^ CSD is a slowly propagating wave of initial depolarization lasting approximately 1 min, moving through the intact brain cortex, regardless of functional division areas, or involving subcortical regions or retina. The initial depolarization is followed by the inhibition of cortical activity for up to 30 min. CSD is initiated by a local elevation in extracellular potassium determining a major disruption of membrane ionic gradients. The efflux of cellular K+ and glutamate is responsible for depolarization of adjacent neurons and glia and increased production of nitric oxide, leading to vasodilation and the transient increase in local blood flow.^6^

Experimental data obtained from animal models suggest that CSD induces inflammatory processes within the brain and meninges which, on their own, increase the firing of first‐ and second‐order trigemino‐vascular neurons.^7^, ^8^, ^9^ In line with these findings, a PET‐MRI study using the ligand ^11^C‐PBR28 reported a strong extra‐axial inflammatory signal in the meninges surrounding the occipital lobe during visual aura in a series of migraine patients.^10^

Calcitonin‐gene‐related peptide (CGRP) is recognized as a relevant player in migraine pathophysiology.^11^ This neuropeptide mediates dural vasodilation and the sensitization of trigeminal nociceptors at the meningeal level and therein contributes to the sensitization of Aδ fibers and the activation of resident glial cells whenever CGRP is released from c fibers in the trigeminal ganglion.^12^, ^13^

Experimental animal findings have also implicated CGRP in spreading depression(SD) involving both the cortex and the retina.^14^, ^15^ Specifically, SD has been reported to elevate the CGRP level in rat cortical slices, as in trigeminal ganglion.^16^ The initial transient arterial dilatation accompanying CSD has been reported to be mediated by CGRP, and in a lesser way by nitric oxide, as suggested by the finding that the inhibition of CGRP receptors prevented CSD induced meningeal vasodilation in cats.^17^

CSD induces the release of CGRP, which acting on its own receptors, can influence cortical or retinal susceptibility to CSD, through a positive loop.^18^, ^19^ The interaction of CGRP with its own receptors could also contribute to facilitating synaptic transmission, via the cAMP‐dependent phosphorylation of N‐methyl‐D‐aspartate(NMDA) receptors.^20^

Recently, it has been reported that multiple, but not single CSD events, significantly increased CGRP mRNA levels and CGRP at 24‐h post‐CSD in the ipsilateral, but not contralateral rat cerebral cortices.^21^ Similarly, a significant elevation of CGRP gene expression was observed in the ipsilateral amygdala at 24‐h postmultiple CSD, but not following a single CSD and in contralateral amygdale.^22^

The induction of CGRP gene expression following repetitive CSD events is sensitive to NR2A regulation at least in amygdala, as suggested by its marked reduction induced by the NR2A‐containing NMDA receptor antagonist, NVP‐AAM077.^23^

Oxidative stress, transient potential ankyrin type receptor TRPA1, and CGRP signaling have been suggested to play pivotal roles in regulating cortical susceptibility to CSD as well as modulating CGRP release in other sites reported to be involved in migraine pathogenesis, such as trigeminal ganglion and the dura mater.^24^, ^25^, ^26^ Certain Src family kinases (SFKs) have been reported fundamental for TRPA1 signaling in CSD; moreover, PKA may serve as an intermediate molecule.^27^

Additionally, in a rat animal model, endogenous CGRP was reported to be released in the cortical tissue, in a calcium‐dependent manner, during reproducible CSD episodes due to repetitive elevations of extracellular potassium concentration. In this model, different CGRP receptor antagonists appeared to have dose‐dependent inhibitory effects on CSD.^18^

Likewise, in an in vitro migraine retinal SD model, a potent antagonist for CGRP receptors, BIBN4096 was able to markedly reduce the magnitude of SD induced by extracellular potassium and also its propagation rate.^14^ In contrast, in another study, CGRP receptor antagonist MK‐8825 dose‐dependently attenuated CSD‐induced trigeminal nerve‐mediated pain response without altering CSD waves and accompanied rCBF response.^28^

The above experimental evidence suggests that CGRP plays a critical role in CSD. If so the potential drugs targeting CGRP might be able to be effective also in migraine aura.

Monoclonal antibodies directed against CGRP, or its receptor (anti‐CGRP‐mAbs)have been established as the first disease‐specific preventive treatment for both episodic and chronic migraines. Data on their effectiveness, as well as their tolerability and safety, emerging from pivotal clinical trials, have been broadly confirmed in real life.^29^, ^30^

Anti‐CGRP‐mAbs have a molecular weight of around 150 kDa, which is an extremely large size compared to CGRP receptor antagonists (gepants) that are in the weight range of 0.2–1 kDa. Because only 0.1%–0.5% of anti‐CGRP‐mAbs cross the intact blood–brain barrier (BBB), the amount of antibody penetrating the barrier during migraine attacks is lower than that required for a significant CGRP inhibition within the brain. Their site of action is therefore outside of the brain, in the dura mater, trigeminal endings, and trigeminal ganglion where they inhibit vasodilation, neurogenic inflammation and neuronal excitability, and facilitation of pain transmission, respectively.^31^

Although BBB is relatively impermeable for anti‐CGRP Mabs, treatment with these drugs may induce modifications in specific brain networks related to trigeminal pain processing with some differences whenever the target of their action is the ligand or the receptor.^32^

Despite the difficulty in accessing cortical and subcortical regions by anti‐CGRP Mabs, a post‐hoc analysis of studies investigating the efficacy and safety of erenumab, along with two clinical reports, have reported a potential efficacy of this class of drugs also for migraine with aura patients.^33^, ^34^, ^35^

In light of the above, the aim of this case series study investigate any changes in the frequency and characteristics of migraine aura attacks due to anti‐CGRP Mabs treatment over 1 year.

## 2 MATERIALS AND METHODS

### 2.1 Patients

Among clinical records of patients treated with anti‐CGRP mAbs (*N* = 148), we retrospectively collected data on 12 migraine patients affected by either migraine with or without aura (3 M and 9 F; 28–57 years old) followed at the Headache Centre of the Neurologic Clinic of the University of Perugia. The frequency of aura episodes ranged from 8 to 14 in the year before administering anti‐CGRP Mab treatment. Migraine patients had been taking anti‐CGRP mAb prophylactic therapy for at least 1 year.

At the beginning of antibody treatment, 4 patients had high‐frequency episodic migraine and 8 complained of chronic migraine. All the 12 patients fulfilled the criteria for reimbursement, according tothe Italian Medicines Agency (AIFA): at least 8 migraine days per month, a Migraine disability Assessment Scale (MIDAS) scoreof at least 11 and the failure to respond, be intolerant or have contraindications to at least three prophylactic drug classes.

Seven patients were treated with erenumab, 2 with fremanezumab, and 3 with galcanezumab.

Symptomatic treatments included: triptans (*n* = 4) nonsteroidal anti‐inflammatory drugs (*n* = 3), and a combination of triptans and nonsteroidal anti‐inflammatory drugs (*n* = 5).

No patients received preventive therapy at the time of inclusion in the anti‐CGRP Mab treatment. Three of the 12 patients had been taking antidepressive drugs (1 venlafaxine, 2 duloxetine) for at least 3 months prior to anti‐CGRP Mab treatment. However, these drugs did not provide any significant benefit for migraine.

The clinical characteristics of the migraine patients are shown in Table 1.

**TABLE 1:** Migraine patients clinical details.

Age	43.7 ± 4.8
M/F	3/9
Disease history	16.2 ± 3.4
Diagnosis at baseline (Pts)	
High‐frequency episodic migraine	4 (33.3)
Chronic migraine	8 (66.7)
Symptomatic drugs used (Pts)	
Triptans	4 (33.3)
NSAIDs	3 (25.0)
Combination of symptomatic drugs	5 (41.7)
Pts with symptomatic drug overuse	10 (83.3)
Anti‐CGRP Mabs used (Pts)	
Erenumab	7 (58.3)
Fremanezumab	2 (16.6)
Galcanezumab	3 (25.0)

Abbreviations: CGRP, calcitonin gene‐related peptide; NSAID, nonsteroidalantiinflammatory drugs; Pts, patients.

*Note*: Values are expressed as mean ± standard error (SE) or number (%).

### 2.2 Clinical data collection

Clinical parameters were recorded for each patient at the scheduled follow‐up times: at baseline, defined as the 3 months before the beginning of treatment, and at each trimester of treatment over the 1‐year treatment period. The study parameters included the number of headache and migraine days/month, the number of days with acute drug intake/month, the frequency of aura episodes, and the scores for MIDAS and for Headache Impact test 6 (HIT‐6).^36^, ^37^ Migraine days per month were recorded from headache diaries whenever a headache fulfilled the criteria for migraine with or without aura.

Aura episodes were also recorded at each follow‐up time. Moreover, the number of aura characteristics including type and duration, headache features, intensity, and duration were registered.

### 2.3 Ethical concerns

Given that this was a retrospective observational study it needed approval from the Internal Hospital Ethics Committee. Informed consent statements were obtained from all patients for the use of their clinical data for scientific purposes. The study conformed to the World Medical Association Declaration of Helsinki.

### 2.4 Statistical analysis

Continuous variables were reported as means ± standard errors of the mean (SE). Categorical variables were reported as absolute numbers and percentages. The normality of continuous variables was tested with the Shapiro–Wilk test which is the most appropriate method for small sample sizes. Because continuous variables did not exhibit a normal/Gaussian distribution, due to the small patient sample size, comparisons were performed among the continuous variables using the nonparametric Mann–Whitney *U* test. A *p* < 0.05 was established as a minimum level of significance.

## 3 RESULTS

In the 3 months before and also during the 1 year of anti‐CGRP Mab treatment, most of the recorded headache episodes were migraine without aura attacks. A significant decrease in mean headache and migraine days per month (reported for each trimester) was observed over the 1 year of treatment, compared with baseline (3 months before) (*p* < 0.0001). A conversion of the chronic form to an episodic form was observed after 3 months in 5 patients starting from the first trimester of treatment.

**FIGURE 1:**
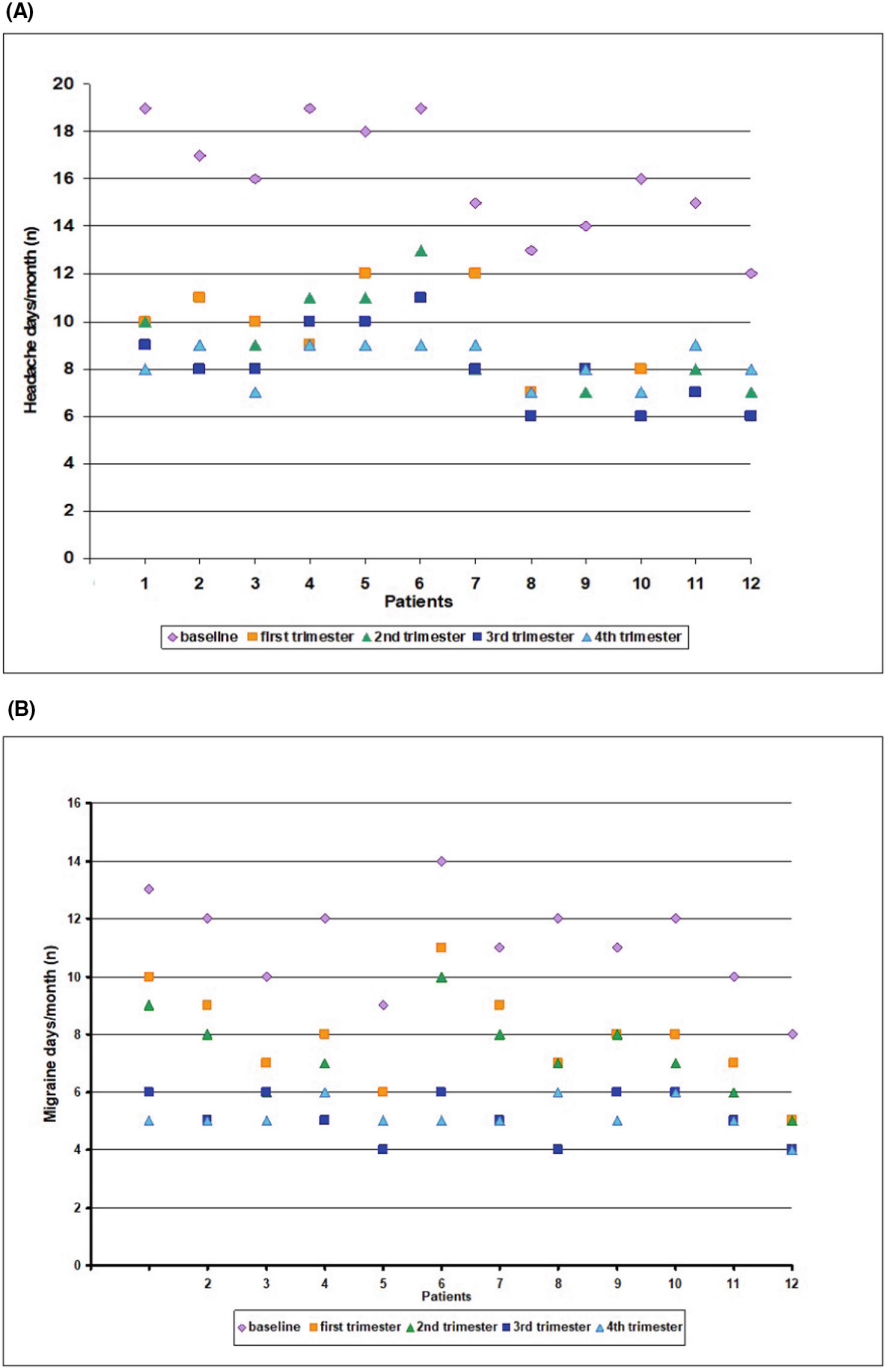
Exact data points indicating headache (A) and migraine days (B) per month of each patient at baseline and each trimester of treatment.

There was a significant reduction in the number of days with acute antimigraine drug intake during anti‐CGRP Mab treatment, compared with the 3 months before treatment (*p* < 0.0001). Furthermore, a reduction in the number of drug abusers was observed, compared with baseline (first trimester *N* = 7, second trimester: *N* = 6, third trimester: *N* = 6, fourth trimester: *N* = 5). The exact data points indicating headache and migraine days per month for each patient at baseline and at each trimester of treatment are shown in Figure 1, respectively.

Statistically significant decreases in MIDAS and HIT6 scores also emerged over the treatment period, compared with the baseline (*p* < 0.0001 and *p* < 0.001, respectively) (Table 2).

**TABLE 2:** Number of headache/migraine days, MIDAS and HIT 6 scores, and migraine with aura details over the 1‐year anti‐CGRP Mabs treatment.

	Baseline §	First trimester	Second trimester	Third trimester	Fourth trimester
Monthly headache days	16.5 ± 0.8	10.2 ± 0.9	9.4 ± 1.1	8.3 ± 0.8	8.7 ± 0.7
Monthly migraine days	11.2 ± 0.9	8.2 ± 0.7	7.2 ± 0.8	5.6 ± 0.7	5.2 ± 0.6
MIDAS	102±	58 ± 10.2	56 ± 9.8	54 ± 8.9	51 ± 8.4
HIT6	68 ± 1.7	62 ± 1.8	59 ± 1.2	55 ± 1.1	56 ± 0.9
Days with acute antimigraine drug intake	13.2 ± 3.9	7.1 ± 2.7	6.4 ± 1.9	5.1 ± 1.5	4.5 ± 1.2
Number of migraine aura episodes	4.16 ± 0.4	3.92 ± 0.7	4.01 ± 0.8	4.01 ± 0.6	4.66 ± 0.8
% of patients presenting migraine aura episodes without headache	1 (16.6%)	3 (25.0%)	4 (33.3%)	3 (25%)	4 (33.3%)
Migraine aura duration (min)	30.8 ± 0.7	25.2 ± 0.6	27.2 ± 0.9	31.3 ± 0.8	35.1 ± 0.9
Type of aura experienced					
Visual	22	18	24	23	28
Sensory	8	12	7	6	5
Both	18	17	17	19	21
Disphasic	2	1	0	1	2
Headache following aura					
TTH like^a^	29 (58.0)	35 (72.9)	41 (80.3)	41 (83.7)	44 (78.5)
Duration of headache accompanying or following migraine aura	9.8 ± 1.1	8.5 ± 1.0	7.2 ± 0.9	7.9 ± 0.7	6.2 ± 0.6

*Note*: Values are expressed as mean ± standard error (SE) or number (%) & referred to the trimester before beginning Anti CGRP treatment.

^a^ All attacks experienced in the course of each trimester.

**FIGURE 2:**
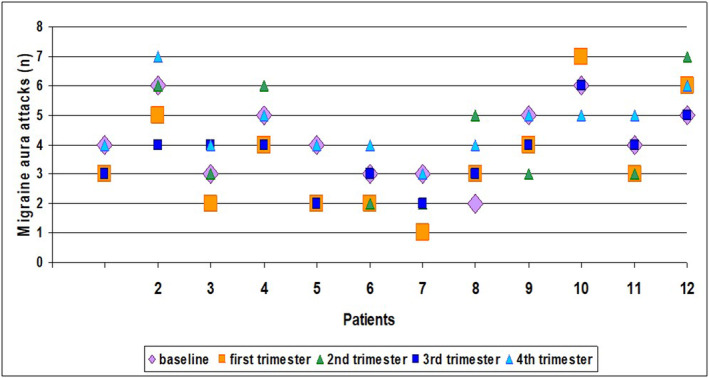
Exact data points indicating the number of migraine aura attacks per month for each patient at baseline and each trimester of treatment.

In contrast, patients continued to complain about migraine with aura attacks despite anti‐CGRP Mab treatment. In fact, the mean number of attacks recorded for each trimester of therapy did not differ significantly from that recorded in the trimester prior to the initiation of treatment. The data indicating the number of migraine aura episodes referred by each patient at baseline and at each trimester of treatment are shown in Figure 2.

Regarding aura attacks without headache, a trend toward an increase in aura attacks was observed over the course of anti‐CGRP Mab treatment. Whenever a headache followed or accompanied by migraine aura, it had tension‐type‐like features in about 2/3 of patients, was of shorter duration compared with the baseline (*p* < 0.01) and was of light or moderate intensity in most cases (Table 2).

## 4 DISCUSSION

Clinical evidence is limited regarding any potential benefit associated with anti‐CGRPMabs as a preventive treatment for both migraine with and without aura. Two case reports have been published supporting the effectiveness of anti‐CGRP Mabs in patients with migraine with aura. Matteo et al.^33^ described the case of 42‐year‐old woman referring chronic migraine with visual aura refractory to various pharmacological treatments who, after the first dose of erenumab 70 mg, presented dramatic drops in both migraine and aura attacks leading to a maximum of one attack per month. This effect persisted for the entire year of treatment. When erenumab was stopped, the frequency of migraine with aura attacks relapsed, returning to the pretreatment level. Likewise, two other patients suffering from migraine with and without aura had a complete disappearance of aura or a reduction in aura duration and intensity over the course of treatment with galcanezumab or erenumab, respectively.^34^

Moreover, results from a post hoc secondary analysis of double‐blind, placebo‐controlled randomized clinical trials conducted in North America, Europe, Russia, and Turkey provided inconclusive results on the effects on migraine aura, despite having shown that the migraine with and without aura patients treated with erenumab 70 or 140 mg had significant reductions in monthly migraine days and monthly acute migraine–specific medications compared with those who received placebo. The authors also stated that a preliminary assessment of aura days was conducted only in chronic migraine patients experiencing aura during baseline and no substantial difference in aura presentation was recorded over the treatment period. Findings on migraine aura patients were considered not definitive due to the limited number of patients available for analysis Furthermore, the authors acknowledged that there may have been selection bias, as almost half of the patients in the analysis had a history of aura and there were diagnostic inaccuracies.^35^

Our study results contrast with the above observations and findings. In our case series, including patients affected by migraine with and without aura treated with anti‐CGRP Mabs over 1 year period, we observed significant reductions in both migraine days without aura per month and the number of days with symptomatic drug intake, both of which were maintained over the course of the treatment period. Accordingly, there were a reduction in disability and an improvement in patient functioning, emerging from significant decreases in the MIDAS and HIT6 scores.

Roughly, 40% of headache attacks recorded at each time point of assessment over the 1‐year treatment period kept the characteristics of migraine according to the ICHD 3 classification, whereas the remaining days exhibited TTH‐like features. In this regard, it is well known that migraine in the chronic or high‐frequency forms generally lose, at least in part, its characteristics. Moreover, migraine preventive drugs, including anti‐CGRP Mabs, may further contribute to altering migraine headache characteristics.

Although there was a decrease in migraine without aura attacks during the 1‐year anti‐CGRP treatment, no changes in the frequency of migraine aura attacks were recorded, compared with the baseline. In about 2/3 of patients, headache following or accompanying aura had tension‐type‐like features and tended to have a shorter duration and lower intensity, compared with attacks occurring before anti‐CGRP Mab treatment. Migraine aura episodes without headache also occurred more frequently during the treatment period, compared with the year before the treatment.

Unlike the case reports cited above, our findings suggest that anti‐CGRP Mabs failed to prevent migraine aura, despite an involvement of CGRP in vascular and neuronal events during CSD.

Specifically, anti‐CGRP Mabs exert their effect mainly peripherally, outside the BBB which is crossed by their large molecules in a small ratio (about 1:1000).^38^ This prevents a significant central effect especially on migraine aura for which there is no clear evidence of a BBB disruption in migraine in general and in migraine aura in particular, as suggested by dynamic contrast‐enhanced high‐field magnetic resonance studies.^39^, ^40^

Preclinical data also suggest that treatment targeting CGRP signaling the BBB passage is not needed for mediating any therapeutic benefits associated with anti‐CGRP Mabs. To this regard, in a glyceryl trinitrate mouse model, the CGRP antagonist olcegepant and the anti‐CGRP Mab ALD405 reduced mechanical sensitivity thresholds, when administered intraperitoneally but not intracerebroventricularly, thus not suggesting a central site of action for both drugs.^41^

In another study, the intravenous injection of fluorescently labeled fremanezumab was able to induce the labeling of dural blood vessels, trigeminal, C2 dorsal root, parasympathetic sphenopalatine, and the sympathetic superior cervical ganglia, but no fluorescent signals were observed in structures within the central nervous system, such as spinal trigeminal nucleus, thalamus, hypothalamus, or cortex. This leads us to hypothesize that anti‐CGRP mAbs might be able to prevent the headache phase of migraine by acting mostly, if not exclusively, outside the brain as the amount of CGRP‐mAbs that enters the brain (if any) is too small to be physiologically impactful.^42^ In line with the above, Melo‐Castillo et al. reported that humanized anti‐CGRP Mab fremanezumab administered intravenously in rats seemed to prevent the activation of high‐threshold but not wide‐dynamic range trigemino‐vascular neurons induced by CSD at spinal and dorsal horn levels.^43^ As the size of the fremanezumab molecule is too large to penetrate the blood–brain barrier, the authors stated that these inhibitory effects could have been secondary to the primary inhibition of peripheral trigemino‐vascular neuron activation.

More recent findings have indicated that the anti‐CGRP Mab fremanezumab does not affect CSD‐induced brief dilatation of the pial arteries, their subsequent prolonged constriction as well as prolonged dilatation of dural arteries. Therein, this further supports that the mechanism of action of fremanezumab in migraine prevention is more likely to be mediated by its ability to reduce or block activation of peripheral nociceptors, rather than by preventing arterial dilatation.^44^ It is plausible that these mechanisms are shared by the other drugs in this class.

To date, anti‐CGRP Mabs does not seem to affect the CSD, which is the pathophysiological substrate of aura, whereas this class of drugs can act upon the neuronal events involving the trigemino‐vascular system activation because of CSD.

Translating these experimental data to our clinical observations, we hypothesize that the aura may not have been affected by anti‐CGRP Mabs, as it might have been suggested by unchanged frequencies in migraine aura attacks over the treatment period. However, the activation of peripheral trigeminal endings and ganglia occurring after CSD may have been influenced by anti‐CGRP Mabs. If so, this may explain why headache following aura in our patients was slightly or moderately intense and shorter in duration over the treatment with anti‐CGRP Mabs, compared with the 3 month period prior. Moreover, the occurrence of migraine aura episodes, not followed by headache over the course of treatment, could have been induced by the peripheral block of trigemino‐vascular activation occurring after CSD, which, in contrast, seems not to be influenced by anti‐CGRP Mabs.

Currently, these observations regarding anti‐CGRP Mabs in relation to aura could be shared by gepants. Recent study results have suggested the effectiveness of the second‐generation gepants rimegepant and atogepant for the preventive treatment of migraine.^50^, ^51^ The main target of gepants is thought to be outside the BBB because only small percentages can be detected in cerebrospinal fluid, compared with plasma, and only supratherapeutic doses are required to reach target receptors protected by the BBB.^45^, ^46^ Nonetheless, experimental evidence suggests that olmegepant administered systemically to mice in vivo can inhibit repetitive CSD events and lead to altering the vascular response to CSD.^47^ These effects can be mediated by their interactions with either CGRP or amylin receptors.^48^, ^49^

In conclusion, future clinical trials and real‐world studies need to be performed to further clarify the role of CGRP‐targeted therapy, not only for preventive treatment of patients with migraine without aura but also for those suffering from migraine with aura.

## AUTHOR CONTRIBUTIONS

All authors contributed to the study conception and design. Material preparation, data collection, and analysis were performed by Elena Cresta, Alessia Bellotti, Giovanni Rinaldi, and Ilenia Corbelli. The first draft of the manuscript was written by Paola Sarchielli and all authors commented on previous versions of the manuscript. All authors read and approved the final manuscript.

## CONFLICT OF INTEREST STATEMENT

The authors have no relevant financial or nonfinancial interests to disclose. The authors have no competing interests to declare that are relevant to the content of this article. All authors certify that they have no affiliations with or involvement in any organization or entity with any financial interest or nonfinancial interest in the subject matter or materials discussed in this manuscript. The authors have no financial or proprietary interests in any material discussed in this article.

## Data Availability

The data that support the findings of this study are available from the corresponding author upon reasonable request.

## References

[1] Headache classification Committee of the International Headache Society (IHS) the international classification of headache disorders, 3rd edition. Cephalalgia. 2018 Jan;38(1):1‐211. doi:10.1177/0333102417738202 29368949

[2] Shah DR , Dilwali S , Friedman DI . Migraine Aura without headache [corrected]. Curr Pain Headache Rep. 2018;22(11):77. doi:10.1007/s11916-018-0725-1 30225597

[3] Yeh WZ , Blizzard L , Taylor BV . What is the actual prevalence of migraine? Brain Behav. 2018;8(6):e00950 4. 30106228 10.1002/brb3.950 PMC5991594

[4] DeLange JM , Cutrer FM . Our evolving understanding of migraine with aura. Curr Pain Headache Rep. 2014;18(10):453. doi:10.1007/s11916-014-0453-0 25230799

[5] Harriott AM , Takizawa T , Chung DY , Chen SP . Spreading depression as a preclinical model of migraine. J Headache Pain. 2019;20(1):45. doi:10.1186/s10194-019-1001-4 31046659 PMC6734429

[6] Lai J , Dilli E . Migraine Aura: updates in pathophysiology and management. Curr Neurol Neurosci Rep. 2020;20(6):17. doi:10.1007/s11910-020-01037-3 32430657

[7] Karatas H , Erdener SE , Gursoy‐Ozdemir Y , et al. Spreading depression triggers headache by activating neuronal Panx1 channels. Science. 2013;339(6123):1092‐1095. doi:10.1126/science.1231897 23449592

[8] Zhang XC , Levy D , Noseda R , Kainz V , Jakubowski M , Burstein R . Activation of meningeal nociceptors by cortical spreading depression: implications for migraine with aura. J Neurosci. 2010;30(26):8807‐8814. doi:10.1523/JNEUROSCI.0511-10.2010 20592202 PMC2907647

[9] Zhang XC , Levy D , Kainz V , Noseda R , Jakubowski M , Burstein R . Activation of central TGV neurons by CSD. Ann Neurol. 2011;69(5):855‐865. doi:10.1002/ana.22329 21416489 PMC3174689

[10] Hadjikhani N , Albrecht DS , Mainero C , et al. Extra‐axial inflammatory signal in Parameninges in migraine with visual Aura. Ann Neurol. 2020;87(6):939‐949. doi:10.1002/ana.25731 32239542 PMC7313427

[11] Edvinsson L . Calcitonin gene‐related peptide (CGRP) is a key molecule released in acute migraine attacks‐successful translation of basic science to clinical practice. J Intern Med. 2022;292(4):575‐586. doi:10.1111/joim.13506 35532284 PMC9546117

[12] Wattiez AS , Sowers LP , Russo AF . Calcitonin gene‐related peptide (CGRP): role in migraine pathophysiology and therapeutic targeting. Expert Opin Ther Targets. 2020;24(2):91‐100. doi:10.1080/14728222.2020.1724285 32003253 PMC7050542

[13] Carneiro‐Nascimento S , Levy D . Cortical spreading depression and meningeal nociception. Neurobiol Pain. 2022;11:100091. doi:10.1016/j.ynpai.2022.100091 35518782 PMC9065921

[14] Wang Y , Li Y , Wang M . Involvement of CGRP receptors in retinal spreading depression. Pharmacol Rep. 2016;68(5):935‐938. doi:10.1016/j.pharep.2016.05.001 27362770

[15] Close LN , Eftekhari S , Wang M , Charles AC , Russo AF . Cortical spreading depression as a site of origin for migraine: role of CGRP. Cephalalgia. 2019;39(3):428‐434. doi:10.1177/0333102418774299 29695168 PMC7007998

[16] Yisarakun W , Chantong C , Supornsilpchai W , et al. Up‐regulation of calcitonin gene‐related peptide in trigeminal ganglion following chronic exposure to paracetamol in a CSD migraine animal model. Neuropeptides. 2015;51:9‐16. doi:10.1016/j.npep.2015.03.008 25998753

[17] Wahl M , Schilling L , Parsons AA , Kaumann A . Involvement of calcitonin gene‐related peptide (CGRP) and nitric oxide (NO) in the pial artery dilatation elicited by cortical spreading depression. Brain Res. 1994;637(1–2):204‐210. doi:10.1016/0006-8993(94)91234-3 8180797

[18] Tozzi A , de Iure A , Di Filippo M , et al. Critical role of calcitonin gene‐related peptide receptors in cortical spreading depression. Proc Natl Acad Sci U S A. 2012;109(46):18985‐18990. 23112192 10.1073/pnas.1215435109 PMC3503217

[19] Russo AF . Calcitonin gene‐related peptide (CGRP): a new target for migraine. Annu Rev Pharmacol Toxicol. 2015;55:533‐552. doi:10.1146/annurev-pharmtox-010814-124701 25340934 PMC4392770

[20] Han JS , Adwanikar H , Li Z , Ji G , Neugebauer V . Facilitation of synaptic transmission and pain responses by CGRP in the amygdala of normal rats. Mol Pain. 2010;6:10. doi:10.1186/1744-8069-6-10 20144185 PMC2829526

[21] Wang Y , Tye AE , Zhao J , et al. Induction of calcitonin gene‐related peptide expression in rats by cortical spreading depression. Cephalalgia. 2019;39(3):333‐341. doi:10.1177/0333102416678388 27919019 PMC5461204

[22] Bu F , Nie L , Quinn JP , Wang M . Sarcoma family kinase‐dependent Pannexin‐1 activation after cortical spreading depression is mediated by NR2A‐containing receptors. Int J Mol Sci. 2020;21(4):1269. doi:10.3390/ijms21041269 32070042 PMC7072958

[23] Bu F , Yuan M , Ma D , Zhu Y , Wang M . Inhibition of NR2A reduces calcitonin gene‐related peptide gene expression induced by cortical spreading depression in rat amygdala. Neuropeptides. 2020;84:102097. doi:10.1016/j.npep.2020.102097 33059243

[24] Shatillo A , Koroleva K , Giniatullina R , et al. Cortical spreading depression induces oxidative stress in the trigeminal nociceptive system. Neuroscience. 2013;253:341‐349. doi:10.1016/j.neuroscience.2013.09.002 24036374

[25] Jiang L , Wang Y , Xu Y , Ma D , Wang M . The transient receptor potential ankyrin type 1 plays a critical role in cortical spreading depression. Neuroscience. 2018;382:23‐34. doi:10.1016/j.neuroscience.2018.04.025 29719223

[26] Jiang L , Ma D , Grubb BD , Wang M . ROS/TRPA1/CGRP signaling mediates cortical spreading depression. J Headache Pain. 2019;20:1‐12. doi:10.1186/s10194-019-0978-z 30841847 PMC6734415

[27] Nie L , Jiang L , Quinn JP , Grubb BD , Wang M . TRPA1‐mediated Src family kinases activity facilitates cortical spreading depression susceptibility and Trigeminovascular system sensitization. Int J Mol Sci. 2021;22(22):12273. doi:10.3390/ijms222212273 34830154 PMC8620265

[28] Filiz A , Tepe N , Eftekhari S , et al. CGRP receptor antagonist MK‐8825 attenuates cortical spreading depression induced pain behavior. Cephalalgia. 2019;39(3):354‐365. doi:10.1177/0333102417735845 28971699

[29] Wang YF , Wang SJ . CGRP targeting therapy for chronic migraine‐evidence from clinical trials and real‐world studies. Curr Pain Headache Rep. 2022;26:543‐554. doi:10.1007/s11916-022-01056-4 35567661

[30] Cohen F , Yuan H , DePoy EMG , Silberstein SD . The arrival of anti‐CGRP monoclonal antibodies in migraine. Neurotherapeutics. 2022;19:922‐930. doi:10.1007/s13311-022-01230-x 35426060 PMC9294119

[31] de Vries T , Villalón CM , MaassenVanDenBrink A . Pharmacological treatment of migraine: CGRP and 5‐HT beyond the triptans. Pharmacol Ther. 2020;211:107528. doi:10.1016/j.pharmthera.2020.107528 32173558

[32] Basedau H , Sturm LM , Mehnert J , Peng KP , Schellong M , May A . Migraine monoclonal antibodies against CGRP change brain activity depending on ligand or receptor target ‐ an fMRI study. Elife. 2022;11:e77146. doi:10.7554/eLife.77146 35604755 PMC9126581

[33] Matteo E , Pensato U , Favoni V , Giannini G , Pierangeli G , Cevoli S . Do anti‐CGRP drugshave a role in migraine aura therapy? J Neurol. 2021;268(6):2273‐2274. doi:10.1007/s00415-021-10546-1 33856547

[34] Albanese M , Mercuri NB . Could the new anti‐CGRP monoclonal antibodies Be effective in migraine Aura? Case reports and literature review. J Clin Med. 2022;11(5):1228. doi:10.3390/jcm11051228 35268319 PMC8911201

[35] Ashina M , Goadsby PJ , Dodick DW , et al. Assessment of Erenumab safety and efficacy in patients with migraine with and without Aura: a secondary analysis of randomized clinical trials. JAMA Neurol. 2022;79(2):159‐168. doi:10.1001/jamaneurol.2021.4678 34928306 PMC8689443

[36] Stewart WF , Lipton RB , Dowson AJ , Sawyer J . Development and testing of the migraine disability assessment (MIDAS) questionnaire to assess headache‐related disability. Neurology. 2001;56(Suppl 1):S20‐S28. doi:10.1212/wnl.56.suppl_1.S20 11294956

[37] Kosinski M , Bayliss MS , Bjorner JB , et al. A six‐item short‐form survey for measuring headache impact: the HIT‐6. Qual Life Res. 2003;12(8):963‐974. doi:10.1023/a:1026119331193 14651415

[38] Felgenhauer K . Protein size and cerebrospinal fluid composition. KlinWochenschr. 1974;52(24):1158‐1164. doi:10.1007/BF01466734 4456012

[39] Hougaard A , Amin FM , Christensen CE , et al. Increased brainstem perfusion, but no blood‐brain barrier disruption, during attacks of migraine with aura. Brain. 2017;140(6):1633‐1642. doi:10.1093/brain/awx089 28430860

[40] Amin FM , Hougaard A , Cramer SP , et al. Intact blood‐brain barrier during spontaneous attacks of migraine without aura: a 3T DCE‐MRI study. Eur J Neurol. 2017;24(9):1116‐1124. doi:10.1111/ene.13341 28727225

[41] Christensen SL , Ernstsen C , Olesen J , Kristensen DM . No central action of CGRP antagonising drugs in the GTN mouse model of migraine. Cephalalgia. 2020;40(9):924‐934. doi:10.1177/0333102420914913 32223300

[42] Noseda R , Schain AJ , Melo‐Carrillo A , et al. Fluorescently labeled fremanezumab is distributed to sensory and autonomic ganglia and the dura but not to the brain of rats with uncompromised blood brain barrier. Cephalalgia. 2020;40(3):229‐240. doi:10.1177/0333102419896760 31856583 PMC7233263

[43] Melo‐Carrillo A , Noseda R , Nir RR , et al. Selective inhibition of Trigeminovascular neurons by Fremanezumab: a humanized monoclonal anti‐CGRP antibody. J Neurosci. 2017;37(30):7149‐7163. doi:10.1523/JNEUROSCI.0576-17.2017 28642283 PMC5546397

[44] Schain AJ , Melo‐Carrillo A , Stratton J , Strassman AM , Burstein R . CSD‐induced arterial dilatation and plasma protein extravasation are unaffected by Fremanezumab: implications for CGRP's role in migraine with Aura. J Neurosci. 2019;39(30):6001‐6011. doi:10.1523/JNEUROSCI.0232-19.2019 31127003 PMC6650995

[45] Sur C , Hargreaves R , Bell I , et al. CSF levels and binding pattern of novel CGRP receptor antagonists in rhesus monkey and human central nervous system: toward the development of a PET tracer. Cephalalgia. 2009;29(Suppl 1):136‐137.

[46] Hostetler ED , Joshi AD , Sanabria‐Bohórquez S , et al. *In vivo* quantification of calcitonin gene‐related peptide receptor occupancy by telcagepant in rhesus monkey and human brain using the positron emission tomography tracer [11C]MK‐4232. J Pharmacol Exp Ther. 2013;347(2):478‐486. doi:10.1124/jpet.113.206458 23975906

[47] Eftekhari S , Kechechyan G , Faas G , Charles A . The CGRP receptor antagonist Olcegepant modulates cortical spreading depression in vivo. Cephalalgia. 2017;37:295‐296.

[48] Hay DL , Walker CS . CGRP and its receptors. Headache. 2017;57(4):625‐636. doi:10.1111/head.13064 28233915

[49] Garelja ML , Walker CS , Hay DL . CGRP receptor antagonists for migraine. Are they also AMY_1_ receptor antagonists? Br J Pharmacol. 2022;179(3):454‐459. doi:10.1111/bph.15585 34076887

[50] Croop R , Lipton RB , Kudrow D , et al. Oral rimegepant for preventive treatment of migraine: a phase 2/3, randomised, double‐blind, placebo‐controlled trial. Lancet. 2021;397(10268):51‐60. doi:10.1016/S0140-6736(20)32544-7 33338437

[51] Ailani J , Lipton RB , Goadsby PJ , et al. Atogepant for the preventive treatment of migraine. N Engl J Med. 2021;385(8):695‐706. doi:10.1056/NEJMoa2035908 34407343

